# Clinical and therapeutic particularities of congenital cataracts in pediatric patients with Down syndrome

**Published:** 2020

**Authors:** Cătălina Ioana Tătaru, Liliana Mary Voinea, Călin Petru Tătaru, George Sima

**Affiliations:** * Department of Ophthalmology, “Carol Davila” University of Medicine and Pharmacy, Bucharest, Romania; Clinical Emergency Eye Hospital, Bucharest, Romania; ** “Carol Davila” Doctoral School, Bucharest, Romania; *** Department of Ophthalmology, “Carol Davila” University of Medicine and Pharmacy, Bucharest, Romania; University Emergency Hospital, Bucharest, Romania; **** Department of Ophthalmology, “Dr. Carol Davila” Central Military Emergency University Hospital, Bucharest, Romania

**Keywords:** congenital cataracts, Down syndrome, congenital cataract, surgical techniques, pseudophakia, postoperative complications

## Abstract

**Objective:** This study aims to identify clinical and therapeutic surgical particularities and postoperative complications encountered in children suffering from Down syndrome and congenital cataract, as well as the existence of a correlation with associated systemic anomalies.

**Methods:** A retrospective interventional study that analyzes cases of congenital cataracts operated on a group of 14 children with Down syndrome, respectively on 26 eyes, was performed. The age of the children at the time of the surgery, the presence of associated ocular and systemic anomalies, the employed surgical technique, the frequency and the type of postoperative complications, were examined.

**Results:** Cataracts present at birth, with recommendations for surgical treatment, were rare among children suffering from Down syndrome, but their frequency increased with age. Most children had systemic anomalies, but also other, usually multiple, ocular anomalies. The rate of postoperative complications was higher than among children with congenital cataracts, but without Down syndrome. In six cases, more than one complication/ case was identified. It was not possible to establish a clear correlation between the number and type of the postoperative complication and the systemic anomalies, nor was it possible to establish a correlation with the functional visual outcomes, because those children had other important ocular anomalies as well.

**Conclusions:** Congenital cataracts with recommendations for surgical treatment in children suffering from Down syndrome have a low incidence, but an increase in frequency can be noticed with age. The recommended surgical technique is the one that involves maneuvers for the prevention of visual axis re-opacification. Per primam implantation is definitely indicated. The risk of postoperative complications is high, in terms of both frequency and number, with the possibility that more than one complication occurs, unrelated to a particular systemic anomaly, in one patient.

## Introduction

Down syndrome or trisomy 21 is one of the best-known genetic syndromes. It is caused by the presence of an additional chromosome 21 in each somatic cell, with a total of 47 instead of 46 chromosomes per cell [**1**]. 

Worldwide, Down syndrome frequency is about 1 case in 700-1,000 live births [**2**] and its occurrence is dependent on multiple socio-cultural factors (birth rate, mother’s age, the possibility of prenatal diagnosis, legality of abortion, etc.) [**3**]. Taking into consideration the frequency worldwide, we estimate that there are approximately 30,000 people with Down syndrome in Romania [**4**]. 

A number of general anomalies are present in patients suffering from Down syndrome: delayed psychomotor development, facial dysmorphia (epicanthic folds, absence of the eyelid groove, upward slanting of the eyes, the so-called mongoloid appearance), heart and gastrointestinal malformations, thyroid dysfunction (hypo- or hyperthyroidism), obstructive sleep apnea, visual disturbances [**5**].

The most common vision problems in children with Down syndrome are: blepharitis, Brushfield spots [**6**], strabismus, nystagmus, various refractive errors, partial or complete congenital cataracts and very rarely congenital glaucoma [**7**]. The association of congenital cataracts with Down syndrome is rare, the existing literature estimating a frequency of 1 per 40,000 live births, but the risk of association increases with age [**2**].

Lens transparency can be affected very rarely from birth, but starting from preschool age, crystalline lens opacities increase [**8**]. Cataracts may progress to forms that require surgery or may require only monitoring in the case of non-evolving crystalline lens opacities.

## Methods

A retrospective interventional study performed at the Clinical Emergency Eye Hospital, Bucharest, Romania, analyzed a group of 14 children, respectively 26 eyes, with operated congenital cataracts and Down syndrome. The same eye surgeon operated those patients between 01/01/2010 and 01/01/2018.

The exclusion criteria were children with Down syndrome and cataracts caused by other factors, namely traumatic cataracts, complicated cataracts, pathological cataracts, congenital cataracts caused by intrauterine infections (rubella, mumps). Systemic and ocular disorders, other than congenital cataracts, were not considered exclusion criteria.

14 children diagnosed with Down syndrome and congenital cataracts with recommendations for surgical treatment, aged between 2 and 17 years, were included in this study. 12 of them presented bilateral cataracts and two, unilateral cataracts.

All patients were operated under general anesthesia by an anesthesiologist with pediatric anesthesia experience, after a careful anamnestic and clinical evaluation and a complete paraclinical examination, in connection with various associated systemic diseases (congenital heart malformation, thyroid dysfunction, obesity, gastrointestinal malformation, obstructive sleep apnea) [**9**].

## Results

Out of the total of 14 children suffering from Down syndrome and congenital cataracts, 10 were males and 4 females, 12 had bilateral cataracts and 2 unilateral cataracts.

The ages the patients were diagnosed with congenital cataracts at, with recommendations for surgical treatment, which was performed (clearing the visual axis by removal of the cataract-affected lens), ranged from 2 to 17 years.

The associated systemic anomalies present in those children with Down syndrome were multiple, a situation that attracted increased attention from the entire medical team. All children presented developmental delay, 8 children - congenital heart malformations, 1 patient - gastrointestinal malformation, 4 patients - thyroid dysfunction (3 patients presented hypothyroidism and one thyroid hyperfunction), 5 patients - obesity and 1 patient - obstructive sleep respiratory dysfunction (**Table 1**).

Ocular anomalies, other than congenital cataracts, were the following: refractive errors [**10**] (20 eyes, of which 2 with severe myopia in 1 patient), nystagmus (8 eyes), strabismus (6 eyes), microphthalmia (1 eye), persistence of Cloquet’s canal (2 eyes) and presence of pupillary membrane (1 eye).

**Fig. 1:**
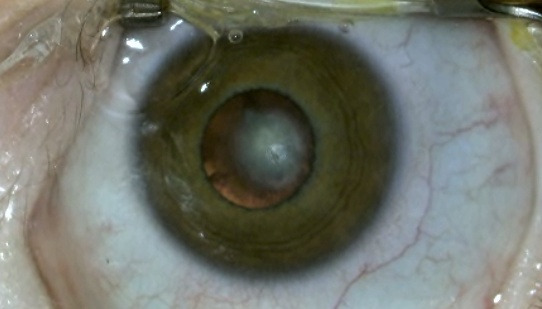
Posterior polar congenital cataract in a patient with Down syndrome from our series

The preoperative ophthalmological examination identified morphopathological forms of congenital cataracts, such as the following: lamellar cataracts – 7 cases, posterior polar cataracts – 3 cases (**Fig. 1**), nuclear cataracts – 6 cases, cerulean cataracts – 4 cases and total cataracts – 6 cases.

**Table 1:** Age, morphopathological forms and associated ocular and systemic anomalies

Case No.	Age	Sex	Morphology	Type	Systemic anomalies	Ocular anomalies
1	2	M	Total	Bilateral	None	OD: persistence of Cloquet’s canal
2	2	M	Total	Bilateral	None	OD: microphthalmia
3	3	M	Nuclear	Bilateral	CHD*	OS: presence of pupillary membrane
4	5	M	Total	Bilateral	gastrointestinal malformation	OS: persistence of Cloquet’s canal
5	5	M	Nuclear	Bilateral	CHD	OU: nystagmus
6	7	M	Nuclear	Bilateral	CHD, hypothyroidism	OU: strabismus
7	11	F	Posterior Polar	Unilateral	CHD	None
8	12	F	Lamellar	Unilateral	Hypothyroidism, obesity	None
9	14	M	Lamellar	Bilateral	Obesity, Sleep apnea	OU: nystagmus, strabismus
10	14	F	Lamellar	Bilateral	CHD, thyroid hyperfunction	None
11	15	M	Cerulean	Bilateral	CHD	OU: nystagmus
12	17	M	Lamellar	Bilateral	CHD, obesity	OU: strabismus
13	17	M	Posterior Polar	Bilateral	Hypothyroidism, obesity	None
14	17	F	Cerulean	Bilateral	CHD, obesity	OU: severe myopia nystagmus
*congenital heart defect						

All 26 surgeries were performed by the same eye surgeon with pediatric cataract surgery experience. The surgical techniques were adapted to each case, depending on the patient’s age, biometric measurements and associated eye anomalies. The chosen surgical treatment was related to the particularities of the children’s eyes: small antero-posterior axis, more curved cornea, special elasticity of the capsular bag, intense inflammatory response to surgical aggressions [**11**].

In all cases, removal of cataract-affected lens, anterior and posterior capsulorhexis were performed, followed by per primam implantation of an IOL in 24 eyes, respectively 13 children (11 children with bilateral cataracts and 2 children with unilateral cataracts), one patient with bilateral cataracts remaining with bilateral aphakia (the patient presented severe myopia and severe horizontal nystagmus).

In this group of 26 operated eyes, 3 types of surgical methods were employed (**Table 2**). In 24 cases, per primam implantation was chosen, depending on the results of the preoperative biometric measurements. 4 eyes of patients aged between 10 and 18 years were operated using a technique consisting in monofocal and toric IOL implantation in the capsular bag, posterior capsulorhexis and limited anterior vitrectomy.

When the integrity of the anterior vitreous allowed (10 operated eyes), a three-piece posterior chamber IOL implantation was preferred, using the optical capture method, without anterior vitrectomy. Using this surgical method, after performing the anterior and posterior capsulorhexis, the IOL haptics were positioned in the sulcus or in the bag, and the optical part in the capsular bag, but behind the posterior capsulorhexis [**12**]. The ages of the children operated by using this technique ranged from 5 to 18 years.

**Fig. 2:**
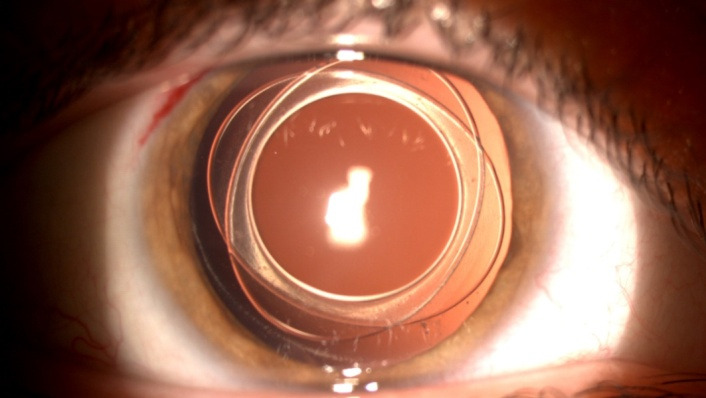
Postoperative image of BIL implant in a 5-year-old patient with Down syndrome – our series

In children aged between 2 and 5 years (5 children, 10 operated eyes), the chosen surgical technique was BIL implantation (bag in the lens, a technique described by Marie-Jose Tassignon) (**Fig. 2**). This technique involves the implantation of a lens with a special design, after performing an anterior and posterior capsulorhexis, equal in size (5 mm), perfectly centered, so that the two remaining crystalline capsules are positioned between the lens haptics [**13**] (bag in the lens, compared to the classic technique – lens in the bag).

In one case, of a 17-year-old child, with severe myopia and nystagmus and already accustomed to wearing glasses, only the removal of the cataract-affected lens was preferred in both eyes, without IOL implantation. Postoperative refraction in this patient was a spherical equivalent = -4D.

All postoperative patients received treatment with antibiotic eye drops and corticosteroid anti-inflammatory drugs for 3 months due to extremely intense postoperative inflammatory reactions. Postoperative complications included visual axis re-opacification – 7 cases (3 BIL cases, 4 non-BIL cases), secondary glaucoma – 7 cases (1 BIL case, 6 non-BIL cases), anterior pseudophakia subluxation – 1 case, severe inflammation – 6 cases (non-BIL), retinal detachment – 1 case (BIL). The described complications occurred in 16 operated eyes, respectively in 9 children. Of these, 7 children also presented systemic anomalies: 4 children with congenital heart malformation, 2 children with thyroid dysfunction and obesity and 1 child with gastrointestinal malformation.

**Table 2:** Surgical technique and postoperative complications

Case No.	Age	Surgical technique	Type	Postoperative complications
1	2	BIL	Bilateral	OS: Visual axis re-opacification
2	2	BIL	Bilateral	OD: Visual axis re-opacification
3	3	BIL	Bilateral	OD: Retinal detachment
4	5	BIL	Bilateral	OD: Severe inflammation, glaucoma
5	5	BIL	Bilateral	None
6	7	Non-BIL	Bilateral	OD: inflammation, glaucoma; OS: visual axis re-opacification
7	11	Non-BIL	Unilateral	OS: Visual axis re-opacification
8	12	Non-BIL	Unilateral	OS: inflammation, glaucoma
9	14	Non-BIL	Bilateral	OS: visual axis re-opacification, OD: inflammation, glaucoma
10	14	Non-BIL	Bilateral	OD: anterior lens dislocation, OS: glaucoma
11	15	Non-BIL	Bilateral	OD: visual axis re-opacification, OS: inflammation, glaucoma
12	17	Non-BIL	Bilateral	None
13	17	Non-BIL	Bilateral	None
14	17	Aphakia	Bilateral	OD: visual axis re-opacification, OS: inflammation, glaucoma

We could notice that 6 cases (eyes) developed more than one postoperative complication and all cases that showed severe inflammation also developed secondary glaucoma.

Visual axis re-opacification is the most common complication in pediatric cataract surgery and can compromise, sometimes completely, the treatment of amblyopia, that is the postoperative functional visual outcome [**14**]. For this reason, posterior capsulotomy is the current standard in congenital cataract surgery, a gesture that can considerably reduce the risk of visual axis re-opacification. Sometimes, anterior vitrectomy performed by employing a bimanual technique at the level of the limbus is necessary, because the remaining capsular epithelial cells can continue to proliferate, although curvilinear and continuous posterior capsulorhexis has been performed.

Postoperative complications encountered in the studied group were compared with data reported in other studies on pediatric patients with and without Down syndrome (**Table 3**) [**15**-**17**].

**Table 3:** Postoperative complications; case series with comparison to literature

Down Syndrome				Without Down Syndrome
Complication	Our Study	Santoro et al. [**15**]	Gardiner et al. [**16**]	Tomkins et al. [**17**]
Visual axis re-opacification	7 (26.9%)	1 (20%)	10 (30.3%)	2 (2.2%)
Secondary glaucoma	7 (26.9%)	3 (60%)	5 (15.15%)	3 (3.3%)
Retinal detachment	1 (3.8%)	1 (20%)	2 (6%)	0
Severe inflammation	6 (23%)			1 (1.1%)
IOL decentration	1 (3.8%)			1 (1.1%)

## Discussions

Congenital cataracts in children with Down syndrome account for 3%-5% of all cases of congenital cataracts. Patients usually have other ocular anomalies as well, which is why these children should be ophthalmologically monitored annually throughout childhood and adolescence, from birth, as recommended by various authors [**18**]. The frequency of congenital cataracts with recommendations for surgical treatment in these children increases with age. In our study, which included 14 patients, none of them was less than 2 years old and most were aged between 5 and 17 years (9 children). The specialized literature estimates the frequency of congenital cataracts associated with Down syndrome to range from <1% [**18**], 5% [**19**] to 50% [**20**]. Our view is that such different reports occur due to the fact that minor crystalline lens opacities, which are the most common crystalline abnormalities in pediatric patients with Down syndrome, are not reported by most authors. Other authors report frequencies of congenital cataracts ranging from 15% to 75%, but based on the study of patients with Down syndrome, regardless of age [**21**]. 

Modern surgical techniques require the circular and continuous capsulorhexis of the anterior and posterior lens capsule, which contributes significantly to reducing the frequency of visual axis re-opacification. The techniques that also use limited anterior vitrectomy for hyaloid removal, a procedure performing IOL optic capture or the BIL technique, provide greater safety against the risk of visual axis re-opacification. BIL technique is sometimes believed to be too difficult as a surgical technique because it requires a calibrated anterior and posterior capsulorhexis, and the using of femtosecond laser assisted capsulectomy facilitates the surgical step of capsulorhexis, and sizing is even more accurate [**13**].

Regardless of age, there are recommendations for per primam artificial lens implantation (in our study, 24 artificial lenses were implanted per primam), because these children have great difficulties in wearing spectacles or contact lenses [**22**]. Most of the authors consulted consider the implantation of a pseudophakia a must when there are no contraindications: congenital glaucoma, microphthalmia, corneal dystrophies, absence of the photomotor reflex. The choice of dioptric power still remains unpredictable, although it is based on increasingly advanced calculation formulas that involve corrections depending on the child’s age [**23**]. In the absence of general anesthesia, biometric measurements in these patients can often be only approximate or even impossible, due to lack of collaboration. When aphakia is chosen as a surgical solution in children under 5 years of age, with bilateral congenital cataracts, per secundam implantation can be considered, and until then the correction is made with spectacles [**24**]. The diopter value of the lenses can be correctly adjusted according to the objective refraction, which changes with age, being able to correct both near and distance vision.

Pseudophakia is the best therapeutic solution for the subsequent treatment of amblyopia, which must be initiated as soon as possible postoperatively [**25**]. In both bilateral and unilateral cataracts, the treatment of amblyopia must be vigorous, sustained and on long term, requiring a good collaboration with the young patient and his/ her family, an aspect difficult to achieve in patients suffering from Down syndrome.

Severe postoperative complications are more numerous than in pediatric patients with congenital cataracts and without Down syndrome, according to data provided by specialized studies (**Table 3**). In the patients we studied, the rate of postoperative complications varied between 3,8% and 26,9%, depending on the type of complication, these percentages being similar to those found in other studies, except for retinal detachment (we identified a very low rate - 3,8%) (**Table 3**). Of the 11 children with severe postoperative complications, 9 also had systemic anomalies. Children with Down syndrome have several health problems that can influence the healing process after surgery. The specialized literature shows an increased rate of complications for other types of surgery, such as cardiovascular surgery, in patients with Down syndrome [**26**].

One of the most important complications is visual axis re-opacification, which might interfere with postoperative visual rehabilitation and could induce deprivation amblyopia. That is the reason why we often perform an anterior vitrectomy. When using the BIL technique, an anterior vitrectomy is not necessary, unless a persistent fetal vascularization is present, because the tight fusing of capsular blades in the interhaptic groove of the artificial lens prevents the escape of the epithelial cells from the capsular bag and their proliferation. If possible, the anterior vitreous membrane should be kept, for it is a barrier between the anterior and posterior segments of the eye.

Secondary glaucoma is the most severe long-term complication. The reported incidence varies, but the risk is higher when the patient is younger, when cataract is associated with other ocular anomalies, severe postoperative inflammations and in the eyes that have been left aphakic [**27**]. In this study, we found 7 eyes with secondary glaucoma, 6 of them presenting aggressive anterior chamber inflammations as well.

Postoperative inflammations are more severe than in adult cataract surgery, and the incidence is higher in pediatric cataract cohort with Down syndrome than without Down syndrome. Inflammation predisposes to visual axis opacification, posterior synechiae, macular edema and secondary glaucoma. In our study, 6 children developed aggressive postoperative inflammation, never as a single complication [**28**].

Increased postoperative complications, influence the functional visual outcome. Complications encountered in this study included visual axis re-opacification, severe inflammation, secondary glaucoma, anterior pseudophakia subluxation and retinal detachment, which required other surgical procedures performed under general anesthesia in these sensitive patients, who frequently have heart anomalies. It was not possible to establish a clear link between the type and number of associated systemic and ocular anomalies and postoperative complications, the group of studied patients being too small to allow such a correlation.

## Conclusions

The association of congenital cataracts with Down syndrome, with recommendations for surgical treatment, is rare, but we believe that the risk of association increases with age. In our study, we did not have any patient under 2 years old. The high frequency in the specialized literature (15%-75%) is based mainly on adult cases. It is possible to improve visual acuity through surgery in children with congenital cataracts and Down syndrome, even if followed by an increased risk of single or multiple postoperative complications. In our study, postoperative complications ranged from 3,8% for retinal detachment and IOL decentration to 26,9% for visual axis re-opacification and glaucoma. Per primam implantation is the best solution for these patients, even at a young age (2 years old), because they have major difficulties in handling contact lenses or glasses. Aphakia may also be an acceptable solution to bilateral cataracts in patients with severe myopia, as it allows them to have a good near vision. The impact on psychomotor development following surgical treatment of congenital cataracts in pediatric patients suffering from Down syndrome may be the subject of future studies.

**Disclosures**

None.
